# Chronic Exposure to HIV-Derived Protein Tat Impairs Endothelial Function via Indirect Alteration in Fat Mass and Nox1-Mediated Mechanisms in Mice

**DOI:** 10.3390/ijms222010977

**Published:** 2021-10-12

**Authors:** Laszlo Kovacs, Thiago Bruder-Nascimento, Lindsey Greene, Simone Kennard, Eric J. Belin de Chantemèle

**Affiliations:** 1 Vascular Biology Center, Augusta University, Augusta, GA 30912, USA; lkovacs@augusta.edu (L.K.); bruder@pitt.edu (T.B.-N.); lindseyGreene@letu.edu (L.G.); skennard@augusta.edu (S.K.); 2 Division of Cardiology, Department of Medicine, Augusta University, Augusta, GA 30912, USA

**Keywords:** HIV Tat protein, endothelial dysfunction, Nox1, leptin

## Abstract

People living with human immunodeficiency virus (HIV) (PLWH) have increased risk for atherosclerosis-related cardiovascular disease (CVD), the main cause of death in this population. Notwithstanding, the mechanisms of HIV-associated vascular pathogenesis are not fully elucidated. Therefore, we sought to determine whether HIV-regulatory protein Tat mediates HIV-induced endothelial dysfunction via NADPH oxidase 1 (Nox1)-dependent mechanisms. Body weight, fat mass, leptin levels, expression of reactive oxygen species (ROS)-producing enzymes and vascular function were assessed in C57BL/6 male mice treated with Tat for 3 days and 4 weeks. Aortic rings and human endothelial cells were also treated with Tat for 2–24 h in ex vivo and in vitro settings. Chronic (4 weeks) but not acute (3 days and 2–24 h) treatment with Tat decreased body weight, fat mass, and leptin levels and increased the expression of *Nox1* and its coactivator *NADPH oxidase Activator 1 (NoxA1)*. This was associated with impaired endothelium-dependent vasorelaxation. Importantly, specific inhibition of Nox1 with GKT771 and chronic leptin infusion restored endothelial function in Tat-treated mice. These data rule out direct effects of HIV-Tat on endothelial function and imply the contribution of reductions in adipose mass and leptin production which likely explain upregulated expression of Nox1 and NoxA1. The Nox1 and leptin system may provide potential targets to improve vascular function in HIV infection-associated CVD.

## 1. Introduction

Currently, more than 37 million people are infected with human immunodeficiency virus (HIV) globally [1]. Successful use of combination antiretroviral therapy (cART) has contributed to a significant reduction in HIV/AIDS-related events [2]. Now people infected with HIV are living much longer, but are exposed to increased risk of non-AIDS-associated chronic illnesses, including cardiovascular disease (CVD) [3]. In fact, atherosclerosis-related CVD is now the leading cause of morbidity and mortality in people living with HIV (PLWH) on cART [4,5,6]. The atherogenesis process is highly complex and involves endothelial dysfunction, which is the initial step in this pathological process [7]. A growing body of evidence indicates that vascular endothelial function is impaired in PLWH [8,9], however, the underlying mechanisms leading to the development of HIV-associated endothelial dysfunction are not completely understood.

Clinical and experimental studies demonstrated that HIV infection itself is related to endothelial dysfunction and atherosclerosis-associated cardiovascular complications. Naïve untreated PLWH possess impaired endothelial-dependent flow-mediated dilation (FMD) of the brachial artery and increased carotid intima–media thickness (cIMT) supporting direct effects of HIV infection on vascular function [10]. Further support to the latter hypothesis has been provided by the observation that HIV elite controllers who have undetectable viral load and are cART-free are exposed to significant atherosclerotic incidents [11,12]. Several HIV-derived proteins, such as Tat, gp120, and Nef have been proposed to be involved in the pathogenesis of endothelial dysfunction that potentially contributes to CVD [13,14,15]. The non-structural HIV-encoded protein Tat is a viral transactivator which is responsible for enhancing the viral transcription and replication [16]. It is actively secreted from HIV infected cells into the extracellular microenvironment and remain in the circulation of PLWH despite the presence of cART [17,18]. Overexpression of Tat has been reported to promote many pathological processes inducing Kaposi’s sarcoma [19], neuropathology [20], and cardiomyopathy [21,22]. However, the impact of Tat on vascular function remain poorly defined.

Endothelial dysfunction is mainly attributable to reduced ability of the endothelium to induce vasorelaxation in response to various stimuli due to reduction in nitric oxide (NO) bioavailability [23,24]. Recent in vitro studies have shown that HIV-derived Tat is associated with oxidative stress, vascular inflammation, increased expression of cell adhesion molecules and reduced expression of endothelial nitric oxide synthase (eNOS), which effects can contribute to NO depletion and consequent impairment of endothelium-dependent vasodilatation. It has been reported that Tat induces oxidative stress and inflammatory mechanisms in brain endothelial cell which may play role in the disruption of the blood–brain barrier (BBB) and lead to HIV-related dementia [25,26,27]. Others have shown that treatment of endothelial cells with Tat resulted in elevated cell surface expression of adhesion molecules, such as intercellular adhesion molecule 1 (ICAM-1), vascular cell adhesion molecule 1 (VCAM-1), and endothelial leucocyte adhesion molecule-1 (ELAM-1), contributing to production of reactive oxygen species (ROS) and endothelial cell activation [28,29,30,31]. Lastly, Tat-incubated endothelial cells from porcine coronary arteries displayed reduced eNOS expression, which was correlated with impaired vascular function [32]. Nevertheless, the molecular mechanisms in which Tat induces endothelial dysfunction remain undetermined in an in vivo setting. In the present study, we tested the hypothesis that the HIV-derived Tat protein induces endothelial dysfunction through alteration in adipose mass and leptin production causing excessive oxidative stress.

## 2. Results

### 2.1. Tat Induces Endothelial Dysfunction

**Figure 1:**
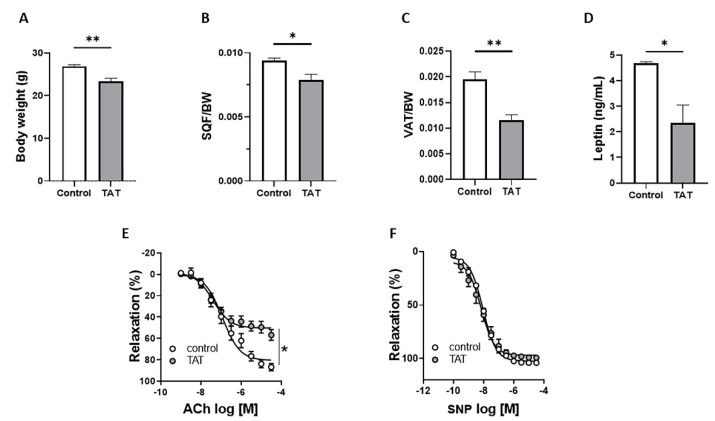
Tat induces endothelial dysfunction. Body weight (**A**), subcutaneous fat depot (SQF/BW; **B**), visceral fat depot (VAT/BW; **C**) plasma leptin levels (**D**), concentration response curves (CRC) to acetylcholine (Ach; **E**) and sodium nitroprusside (SNP; **F**) in aortic rings from control (vehicle-treated) and TAT-treated mice (TAT, 3.2 µg/kg per day for 4 weeks, i.p.). Data are presented as mean ± SEM. *n* = 5–6; * *p* < 0.05; ** *p* < 0.01 vs. Ctrl.

In order to investigate whether Tat mediates HIV-associated endothelial dysfunction, male mice were submitted to 4 weeks of Tat treatment. We found that chronic treatment with Tat markedly reduced body weight (Figure 1A), subcutaneous (Figure 1B), visceral adipose tissue mass (Figure 1C), and plasma leptin levels (Figure 1D) in WT mice. In addition, we conducted concentration response curves (CRC) to acetylcholine (Ach) and sodium nitroprusside (SNP) in aortic rings from control and Tat-treated mice to examine the effects of Tat on vascular function. Tat-treated mice showed a significant reduction in Ach-mediated relaxation (Figure 1E), but no alteration in SNP-mediated vasodilatation (Figure 1F), compared with vehicle-treated (control) mice indicating impaired endothelium-dependent relaxation.

### 2.2. Tat Does Not Have Direct Effect on Endothelial Function

**Figure 2:**
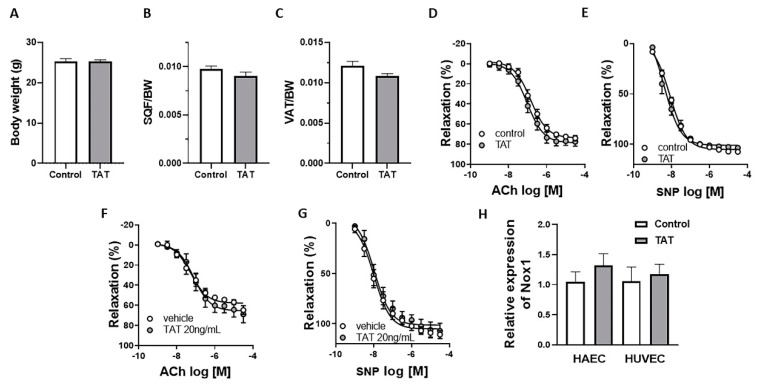
Tat does not have direct effect on endothelial function. Body weight (**A**), subcutaneous fat depot (SQF/BW; **B**), visceral fat depot (VAT/BW; **C**), concentration response curves (CRC) to acetylcholine (Ach; **D**) and sodium nitroprusside (SNP; **E**) in aortic rings from control (vehicle-treated) and TAT-treated mice (TAT, 3.2 µg/kg per day for 3 days, i.p.). CRC to acetylcholine (Ach; **F**) and sodium nitroprusside (SNP; **G**) in the presence of vehicle or Tat protein (20 ng/mL) in aortic rings from control mice. Gene expression of *Nox1* (**H**) in vehicle (control) and Tat-treated (20 ng/mL for 24 h) HAEC and HUVEC cells. Data are presented as mean ± SEM. *n* = 5–6.

To investigate whether Tat alters endothelial function via indirect metabolic derangements or direct vascular effects, male mice were treated with Tat for 3 days and aortic rings from control mice were exposed to Tat for 2 h. As shown in Figure 2, short-term administration of Tat did not reduce body weight (Figure 2A), nor subcutaneous (Figure 2B) and visceral adipose tissue mass (Figure 2C). More importantly, endothelium-dependent relaxation (Figure 2D) and endothelium-independent relaxation (Figure 2E) remained intact in 3-day Tat-treated mice. Similarly, acute ex vivo treatment of aortic rings and in vitro incubation of human aortic endothelial cells (HAEC) and human umbilical vein endothelial cells (HUVEC) with Tat did not impaired vascular function (Figure 2F–H). Together, these data suggest that acute treatment with Tat is not sufficient to induce endothelial dysfunction supporting the indirect effect of Tat on vascular function potentially via reduction in fat mass depots.

### 2.3. Tat-Induced Endothelial Dysfunction Is Mediated by Nox1

**Figure 3:**
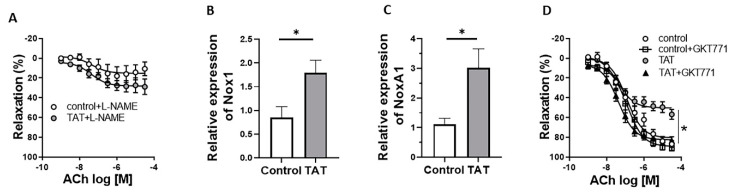
Tat-induced endothelial dysfunction is mediated by Nox1. Concentration response curves (CRC) to acetylcholine (Ach) in aortic rings in the presence of L-NAME (100 μmol/L) (**A**) or GKT771 (10 μmol/L) (**D**) and gene expression of *Nox1* (**B**) and *NoxA1* (**C**) in thoracic aorta from control (vehicle-treated) and TAT-treated mice (TAT, 3.2 µg/kg per day for 3 days, i.p.). Data are presented as mean ± SEM. *n* = 5–6; * *p* < 0.01 vs. Ctrl.

To examine whether decreased activation of endothelial nitric oxide synthase (eNOS) has a role in the Tat-induced endothelial dysfunction, aortic rings from control and chronic Tat-treated mice were pretreated with NOS-inhibitor N (ω)-nitro-L-arginine methyl ester (L-NAME) followed by measurements of relaxation responses to ACh. We found that L-NAME significantly reduced ACh-mediated relaxation in both control and Tat-treated mice indicating the NO dependence of vasodilatation in the aorta and reduction in NO bioavailability in Tat treated animals (Figure 3A).

In order to determine the potential mechanism leading to decreases in NO bioavailability and vascular function, we measured the expression of the ROS-generating enzyme *NADPH oxidase 1 (Nox1)* and of its coactivator (*NoxA1*) in aorta tissues from control and chronic Tat-treated mice. As shown in Figure 3B,C, Tat markedly increased the aortic expression of *Nox1* and its coactivator *NoxA1*, respectively. To investigate the role of Nox1 in Tat-induced endothelial dysfunction, relaxation responses to ACh were assessed in the presence and absence of specific Nox1 inhibitor GKT771 in aortic rings. Inhibition of ROS production with GKT771 fully restored endothelial function in Tat-treated mice (Figure 3D) demonstrating the mediating role of Nox1 in the endothelial dysfunction evoked by Tat.

### 2.4. Leptin Restores Endothelial Function in Tat-Treated Mice

**Figure 4:**
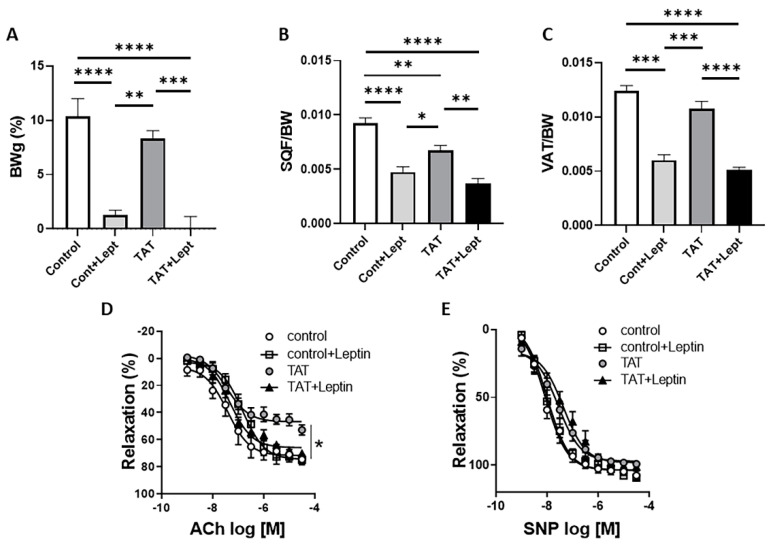
Leptin restores TAT-induces endothelial dysfunction in mice. Body weight (**A**), subcutaneous fat depot (SQF/BW; **B**), visceral fat depot (VAT/BW; **C**), concentration response curves to acetylcholine (Ach; **D**) and sodium nitroprusside (SNP; **E**) in aortic rings from control (vehicle-treated) and TAT-treated mice (TAT, 3.2 µg/kg per day for 4 weeks, i.p.) in the presence or absence of leptin treatment (0.3 mg/kg per day for 7 days, via osmotic minipump). Data are presented as mean ± SEM. *n* = 5–6; * *p* < 0.05; ** *p* < 0.01; *** *p* < 0.001, and **** *p* < 0.0001 vs. Ctrl.

As we reported in Figure 1, chronic Tat treatment significantly decreased fat mass and plasma levels of adipokine leptin in WT mice (Figure 1B–D). To investigate the impact of reduced fat mass and leptin levels on the vascular function, we assessed whether restoration of leptin levels via chronic infusion restores Tat-induced endothelial dysfunction. After 3 weeks of Tat exposure, mice were treated with leptin (0.3 mg/kg/day) or vehicle for 7 more days. Chronic leptin administration restored endothelial function (Figure 4D,E) in Tat-treated mice in spite of further decreasing body weight and fat mass (Figure 4A–C) suggesting the direct action of leptin on the endothelium. This indicates that reduction in leptin signaling but not fat mass promotes endothelial dysfunction in Tat-treated mice.

## 3. Discussion

A growing body of evidence indicates that HIV infection per se is an independent risk factor for endothelial dysfunction and atherosclerosis-associated CVD [33,34,35,36,37]. In the present study, we investigated the molecular mechanism whereby HIV-derived Tat protein impairs endothelial function. We demonstrated, for the first time to our knowledge, that HIV protein Tat plays a critical role in the viral infection-induced endothelial dysfunction via elevation of Nox1 expression and reduction in adipokine leptin secretion.

Lipodystrophy, the metabolic disorder characterized by alteration in fat mass content and distribution, is a key feature of HIV and correlates with the development of endothelial dysfunction and atherosclerosis [38,39,40]. Furthermore, lipoatrophy is associated with low serum level of adipokine leptin in PLWH [41]. Clinical and experimental studies have shown that viral infection and HIV-encoded proteins contribute to the pathological process of lipodystrophy [42,43,44,45]. It has been reported that HIV-1 Tat protein not only impairs adipogenesis, but also promotes apoptosis of adipocytes resulted in decreased fat mass [46,47,48,49]. However, the link between viral infection-related lipoatrophy and impaired vascular function, as well as the signaling mechanisms by which viral infection stimulates lipodystrophy is not well defined. In agreement with the recent studies, our present work demonstrated that HIV-derived Tat significantly decreased fat mass and leptin production. Importantly, reduced adipose mass and leptin levels were associated with diminished vasorelaxation to acetylcholine, a hallmark for endothelial function, but not with the changes in smooth muscle cell-dependent vasodilatation (SNP) which suggests that viral protein Tat impairs endothelial function specifically.

In opposition to our present findings, previous in vitro study in isolated porcine arteries reported that Tat directly alters endothelial function [32]. Here, we postulate the indirect impact of Tat on vasculature and propose the mechanism by which Tat-induced reduction in fat mass contributes to the endothelial dysfunction. Notably, short-term (3 days) exposure to Tat did not cause changes in body weight and fat mass and importantly did not alter vascular function in mice. Moreover, we demonstrated that Tat-treated arteries from control mice exhibited normal vascular function. ROS production by Nox1 is the main cause of NO depletion and consequent endothelial dysfunction [50]. With in vitro experiments, we found that HAEC and HUVEC cells treated with Tat did not show increased gene expression of Nox1 indicating normal redox status. Altogether, these data provide convincing evidence that endothelial dysfunction observed in the chronic Tat-treated mice attributable to the decreased adipose tissue and not originates from the direct effects of the HIV-derived Tat on the endothelium.

The regulatory Tat protein not only facilitates the transcription of HIV, but it is also implicated in the pathogenesis of endothelial dysfunction and atherosclerosis-associated CV complications in PLWH [51]. In contrast to our results supporting indirect effects of Tat on endothelial function mediated via leptin reduction, others have reported that HIV protein Tat stimulates oxidative stress by increasing ROS production and decreasing antioxidant capacity [52,53,54,55]. Many have shown association between the HIV-encoded Tat and NADPH-oxidases. Wu and colleagues reported increases in the activation of Nox2 and Nox4 in Tat-treated HUVEC via Rac1-dependent mechanism contributing to cytoskeletal rearrangements and cell proliferation/survival, respectively [56]. Other study has demonstrated that PI3K/Akt signaling is implicated in the Tat-induced Nox2-dependent ROS production in multinuclear activation of galactosidase indicator (MAGI) cells leading to the long terminal repeat region (LTR) transactivation [57]. Youn et al. have also shown that HDAC6 mediates the Tat-induced Nox2 activation and inflammatory responses in astrocytes [58]. The discrepancy between the latter findings and the present study can likely be explained by several factors. A common point between all these studies is their in vitro nature. In addition, the applied dose of Tat was considerable higher in many and analogos in few studies in comparison with ours. Moreover, the duration of the Tat treatment and origin of the cells were quite different among these experimentations. Here, we claimed indirect relationship between Tat and Nox1-mediated impairment of vascular function by the fact that chronic Tat treatment, but not acute treatment, promoted the expression of ROS-producing enzymes Nox1 and its coactivator and caused endothelial dysfunction. Importantly, these pathological processes were associated with a reduction in adipose mass and leptin levels. These findings are in concert with our recently published paper, which clearly demonstrated that increased Nox1 expression and ROS generation is involved in the HIV protease inhibitor ritonavir-induced endothelial dysfunction through reducing the leptin signaling [59]. In addition, we also showed that inhibition of Nox1 restored the deficiency in endothelial function evoked by Tat. Altogether, our studies herein confirmed the indirect role of Tat on the ROS-dependent HIV-associated endothelial dysfunction.

The role of leptin in the pathogenesis of CVD remains controversial. Many studies have demonstrated that elevated leptin levels contribute to development of vascular dysfunction and CV events [60,61,62]. In contrast, we previously reported that mice with congenital generalized lipodystrophy (CGL) and mice treated with HIV protease inhibitor ritonavir exhibit endothelial dysfunction due to reduced leptin secretion, and leptin supplementation strikingly restored endothelial function [59,63]. It is also known that the major source of leptin is the subcutaneous fat depot [64]. Here, we showed that HIV-encoded Tat significantly decreased subcutaneous fat mass in mice. In order to test whether subcutaneous fat mass reduction impairs endothelial function via lowering leptin levels, Tat-treated mice were chronically infused with leptin for 7 days. In concert with our previous work, we demonstrated that chronic leptin administration was able to restore endothelial function, indicating the regulatory role of leptin signaling in the HIV viral Tat protein-induced endothelial dysfunction. However, a limitation of this study is the lack of evidence, which clearly shows that leptin restores endothelial function in Nox1-dependent manner. Further studies are needed to investigate direct molecular links between leptin and Nox1.

Increased levels of fat mass is a leading risk factor for CVD in non-infected people [65]. Although, current PLWH on cART develop obesity [66], interestingly the increases in adiposity are not associated with further risk for development of CV event in the HIV infected population. Notably, Koethe and colleagues reported that obesity in PLWH did not adversely affect the circulating plasma levels of vascular adhesion molecules, or measurements of carotid intima-media thickness (cIMT) or brachial artery flow-mediated dilation (FMD) [67]. In addition, recent study demonstrated that increases in body mass index (BMI) were not related to greater risk of CVD in PLWH on cART [68]. More importantly, plasma concentrations of leptin has been shown to be higher in patient on cART and increased levels of leptin was positively correlated with FMD and negatively with cIMT [69]. In agreement with this observation, our findings established the importance of leptin signaling in the protection of vascular function and health in PLWH.

## 4. Materials and Methods

### 4.1. Animals

The 10 week-old male C57BL/6 mice were purchased from Jackson Laboratory. All animals were housed in an American Association of Laboratory Animal Care-approved animal care facility at Augusta University. Animals were housed at ambient temperature with 12:12 h light–dark cycles with food and water ad libitum. All procedures and protocols were approved by the Augusta University Institutional Animal Care and Use Committee (IACUC protocol #2011-0108) and are compliant with guidelines set forth by the NIH.

### 4.2. Human Aortic Endothelial Cells (HAEC) and Human Umbilical Vein Endothelial Cells (HUVEC)

In order to analyze whether Tat regulates Nox1 expression, HAEC and HUVEC cells (Lonza, Walkersville, MD, USA) were stimulated with Tat (20 ng/mL, NIH HIV Reagent Program) for 24 h. After that, *Nox1* gene expression was analyzed by quantitative real-time RT-PCR as described below.

### 4.3. Treatments

Mice were treated with saline or Tat (3.2 µg/kg per day for 4 weeks) by intraperitoneal (i.p.) injection. The concentrations of Tat was chosen based on the study by Xiao et al. who reported that estimated Tat serum levels in PLWH is between 2 and 40 ng/mL [18]. After 3 weeks of Tat treatment, mice were separated into 2 groups and treated with either leptin (0.3 mg/kg per day, 0.417 μg/h, ProSpec, Ness-Ziona, Israel) or vehicle via subcutaneous osmotic mini-pumps (ALZET, Cupertino, CA, USA; model 1007D, 0.5 μL/h) for 7 more days, as previously described by [59]. A short-term Tat treatment (3.2 µg/kg per day for 3 days, NIH HIV Reagent Program) was also used to examine the potential direct effects of Tat on endothelial function.

### 4.4. Metabolic Characterization

Body weight was measured at baseline and at the end of the treatment. After 4 weeks of treatment, mice were anesthetized (isoflurane 5%) and euthanized via decapitation, in accordance with our approved animal protocol. Trunk blood was collected for plasma isolation to determine the plasma leptin levels using ELISA kit from Millipore Sigma (Burlington, MA, USA). Subcutaneous (SQF) and visceral adipose tissue (VAT) were collected and weighed.

### 4.5. Vascular Function Studies

Thoracic aortas were excised, cleaned of adipose tissue, cut in four 2 mm rings and mounted on a DMT wire myograph (Ann Arbor, MI, USA) as previously described [59,62]. Concentration response curves (CRC) to acetylcholine (Ach, Sigma-Aldrich, St. Louis, MO, USA); (0.1 nmol/L to 100 μmol/L) and sodium nitroprusside (SNP, (Sigma-Aldrich, MO, USA); (0.1 nmol/l to 10 μmol/L) in the presence or absence of the nitric oxide synthase (NOS) inhibitor Nω-Nitro-L-arginine methyl ester hydrochloride (L-NAME, 100 μmol/L, Sigma-Aldrich, MO, USA) or specific Nox1 inhibitor GKT771 (10 μmol/L; Genkyotex, Geneve, Switzerland) were performed and recorded with the LabChart^®^ analysis software (AD Instruments^®^, Colorado Springs, CO, USA). Aorta rings were also incubated with 20 ng/mL Tat protein for 2 h followed by CRC to ACh and SNP. CRCs to ACh and SNP are presented as percent of 5HT-induced constriction. The individual CRCs were fitted by non-linear regression analysis. pD2 (defined as the negative logarithm of the EC50 values) and maximal response (Emax) were determined.

### 4.6. Real-Time PCR

The aorta were homogenized, then RNA was isolated (Trizol Plus, Invitrogen, Carlsbad, CA, USA) and the concentration was established with a NanoDrop 1000 (NanoDrop Technologies, Wilmington, DE, USA). cDNA was generated by reverse transcription using SuperScript III (Thermo Fisher Scientific, Newington, NH, USA) and real-time quantitative RT-PCR was performed with SYBR-Green Supermix (Applied Biosystems, Foster City, CA, USA). Genes analyzed were NADPH oxidase 1 (Nox1), NADPH oxidase Activator 1 (NoxA1) and glyceraldehyde 3-phosphate dehydrogenase (GAPDH), which was used as housekeeping gene. The sequence of the primers were the following: forward (FW)-5′CATGGCCTGGGTGGGATTGT3′ and reverse (RV)-5′TGGGAGCGATAAAAGCGAAGGA3′ for Nox1; FW-5′ACGGTGGATGTTCTGTGTGA 3′ and RV-5′AAGCATGGCTTCCACATAGG3′ for NoxA1; FW-5′ACCCAGAAGACTGTGGATGG3′, and RV-5′ACATTGGGGGTAAGGAACAC3′ for GAPDH. Relative gene expression (2−ΔΔCt) was calculated as we previously described [70].

### 4.7. HIV-Derived Tat Protein

HIV-derived Tat protein (ARP-2222) was obtained through the NIH HIV Reagent Program. ARP-2222 is a full length, biologically active recombinant protein derived from HIV-1 IIIB Tat protein. This regulatory protein was produced in an *E. coli* expression system and purified by affinity chromatography on heparin sepharose, followed by reverse phase chromatography.

### 4.8. Statistical Analysis

Results are shown as means ± SEM for n experiments. *p* < 0.05 was considered significant. Differences in means between 2 groups for nonrepeated variables were compared by unpaired Student t test. Differences in means among groups and treatments were compared by 2-way ANOVA with repeated measures, when appropriate. Tukey test was used as the post hoc test (GraphPad).

## 5. Conclusions

**Figure 5:**
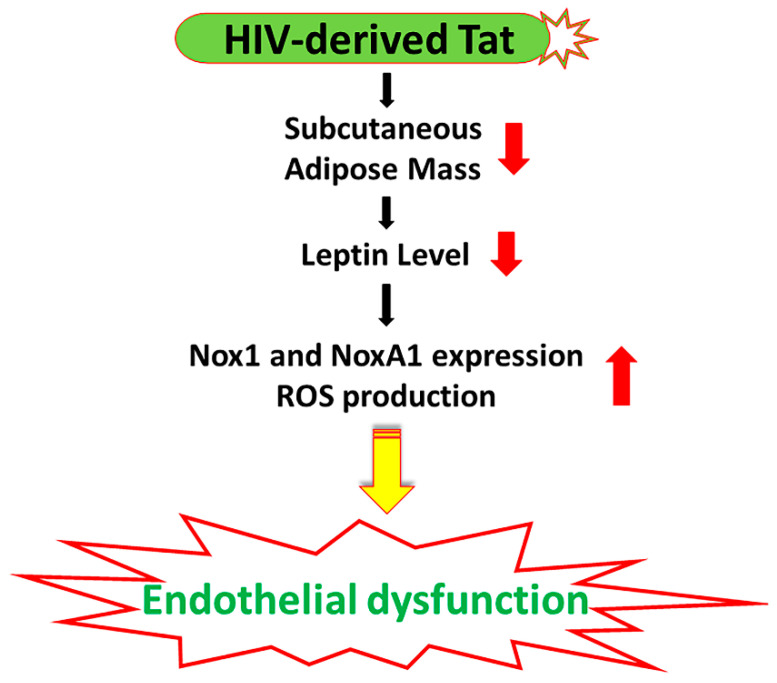
Schematic illustrating the possible mechanisms whereby HIV-derived protein Tat reduces adipose mass and plasma leptin levels leading to increased Nox1 and NoxA1 expression and ROS production and ultimately contributes to endothelial dysfunction.

In conclusion, using an in vivo approach, our study demonstrated that the HIV transactivator protein Tat contributes to HIV-associated endothelial dysfunction via promoting adipose mass loss, and leptin level reduction leading to an upregulated expression of Nox1 and NoxA1 (Figure 5). Targeting the Nox1 and leptin signaling might be an attractive therapeutic approach for CV disorders in PLWH.

## Data Availability

The data presented in this study are available on request from the corresponding author.
